# Case report: Asp194Ala variant in MFN2 is associated with ALS-FTD in an Italian family

**DOI:** 10.3389/fgene.2023.1235887

**Published:** 2023-07-20

**Authors:** C. Vinciguerra, A. Di Fonzo, E. Monfrini, D. Ronchi, S. Cuoco, G. Piscosquito, P. Barone, M. T Pellecchia

**Affiliations:** ^1^ Center for Neurodegenerative Diseases (CEMAND), Department of Medicine, Surgery and Odontology “Scuola Medica Salernitana”, University of Salerno, Salerno, Italy; ^2^ Dino Ferrari Center, Department of Pathophysiology and Transplantation, University of Milan, Milan, Italy; ^3^ IRCCS Fondazione Ca’ Granda Ospedale Maggiore Policlinico, Neurology Unit, Milan, Italy

**Keywords:** mitofusin 2-gene (MFN2), amyotrophic lateral sclerosis (ALS), frontotemporal dementia (FTD), whole exom sequencing, charcot marie tooth (CMT)

## Abstract

**Background:** *MFN2* gene encodes the protein Mitofusin 2, involved in essential mitochondrial functions such as fusion, trafficking, turnover, and cellular interactions. We describe a family carrying a novel *MFN2* mutation associated with ALS-frontotemporal dementia (FTD) clinical phenotype in the mother and Charcot-Marie-Tooth disease type 2A (CMT2A) in her son.

**Case presentation:** The mother, a 67-year-old woman, referred to us for a three year-history of mood disturbance and gait impairment, and a more recent hypophonia, dysarthria, dysphagia, and diffuse muscle wasting. Family history was positive for psychiatric disorders and gait disturbances. Brain 18F-FDG PET showed severe hypometabolism in the fronto-temporal brain cortex bilaterally. Electrodiagnostic studies (EDX) showed severe motor axonopathy in the bulbar, cervical and lumbosacral districts. Her 41-year-old son had a history of mood depression and sensory disturbances in the limbs, along with mild muscle wasting, weakness, and reduced reflexes. Nerve conduction studies revealed a moderate sensory-motor polyneuropathy, while brain MRI was normal. Whole exome sequencing of the patients’ DNA identified the novel *MFN2* (NM_014874.4) variant c.581A>C p.(Asp194Ala).

**Conclusion:** Our findings provide evidence of heterogenous clinical manifestations in family members sharing the same *MFN2* molecular defect. Additionally, we present the first documented case of ASL-FTD associated with an *MFN2* mutation, thereby expanding the range of MFN-related disorders. Further research involving larger cohorts of patients will be needed to better understand the role of *MFN2* as a contributing gene in the development of ALS-FTD.

## Introduction

Mitofusin 2 (MFN2) is a mitochondrial transmembrane GTPase protein involved in several mitochondrial activities (fusion, trafficking, turnover, contacts with other organelles), the balance of which results in the appropriate mitochondrial shape, function, and distribution within the cell (Chen et al., 2003).

Mutations of *MFN2* gene have recently been identified as the cause of approximately one-third of dominantly inherited cases of the axonal Charcot-Marie-Tooth disease (CMT type 2A) and of rarer clinical variants, including a severe, early-onset axonal neuropathy, in some instances associated with pyramidal tract involvement (CMT type 5), optic atrophy (CMT type 6), and, occasionally, alterations of cerebral white matter (Verhoeven et al., 2006; Pareyson et al., 2013). Interestingly, one patient with co-occurrence of ALS and Charcot-Marie-Tooth disease type 2A associated with a novel mutation in the *MFN2* gene has been also described, but this co-occurrence was supposed to be casual rather than causal (Marchesi et al., 2011). More recently, in a mouse model of ALS, MFN2 protein has been found to interact in complex with Transactive response DNA-binding protein of 43 kDA (TDP-43), involved in frontotemporal lobar degeneration (FTLD) and ALS (Davis et al., 2018).

Here, we report a family carrying a novel *MFN2* mutation associated with an ALS-FTD phenotype in the mother and a CMT2A in the son.

## Case presentation

The mother is a 67-year Caucasian woman presenting with a 3-year history of depression, apathy and recent occurrence of gait difficulties with frequent falls. Family history was positive for psychiatric disorders and gait disturbances. In particular, one sister suffering from depression and unspecified motor difficulties, died at 56 years without receiving any neurological diagnosis, and a maternal cousin had a mood disorder and committed suicide.

**FIGURE 1:**
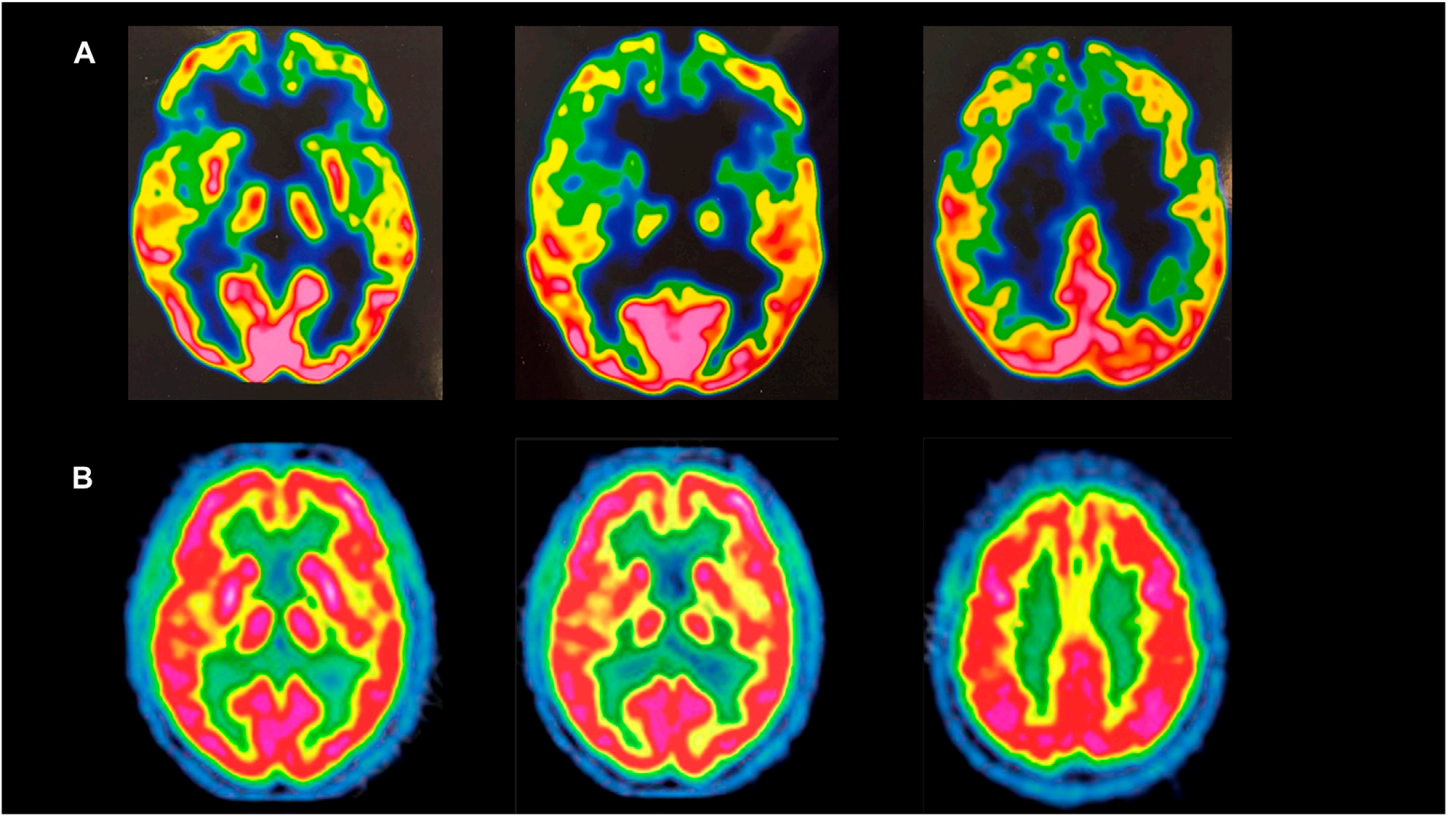
**(A)** Brain 18-FDG PET of the mother, showing severe hypometabolism in the fronto-temporal brain cortex bilaterally. **(B)** Brain 18-FDG PET of a healthy subject.

Neurological examination showed tongue fasciculations, pseudobulbar features, hypophonia, dysarthria, dysphagia, spastic paraparetic gait with bilateral stepping (she needed assistance to walk), brisk upper limb and absent lower limb deep tendon reflexes, bilateral Hoffman and Babinski signs, severe diffuse muscle wasting, upper and lower limb weakness, especially in the distal districts, with complete loss of ankle dorsiflexion and plantar flexion. No sensory disturbances were detected. A 3-Tesla brain MRI revealed bilateral atrophy of the frontal lobes. Brain 18F-FDG PET showed severe hypometabolism in the fronto-temporal brain cortex bilaterally (Figure 1).

A comprehensive neuropsychological assessment showed low Mini Mental State Examination scores (22/30), associated to a dysexecutive syndrome. Specifically, the spatial-temporal orientation and the autobiographical memory were preserved, except for details of some relatives’ information. The insight was conserved. The speech was repetitive and dysarthric. Short-term visual memory was at the lower limit of the norm. Regarding executive and attentional domain, she showed inhibitory control deficit, sensitivity to interference, impairment on go-no go tasks, planning and attentional shift deficits.

Electrodiagnostic studies (EDX) showed marked reduction or absence of compound muscle action potential (CMAP) amplitudes in all motor nerves examined (right and left tibial, peroneus and ulnar nerves). Sensory nerve conduction study was normal.

Standard needle EMG examination, performed in the bulbar, thoracic regions and at least two proximal and distal muscles innervated by different roots and peripheral nerves for each limb, revealed widespread active positive sharp waves and fasciculation potentials in all districts, associated to chronic motor unit changes. Motor evoked potentials (MEPs), measured from posterior tibial nerves, resulted absent with adequate distal CMAP values, suggesting a cortical inexcitability. A diagnosis of definite ALS was made according revised El Escorial criteria (Ludolph et al., 2015). She died 1 year later due to aspiration pneumonia.

Her son is a 41-year-old Caucasian man with a history of mood depression, diffuse limb numbness, tingling and burning in the distal upper and lower limbs for at least 4 years. For the latter disturbances, initially prevalent on the right hand, he underwent decompression surgery of the right median nerve at wrist, reporting no improvement. He also had a diagnosis of obsessive-compulsive disorder since the age of 25 years and is currently treated with sertraline, 50 mg/day.

At admission to our department, neurological examination showed abnormal sensation in the distal lower limbs, mild muscle wasting and weakness on the distal upper and lower limbs with generalized hyporeflexia.

Electrophysiological study revealed a moderate sensory-motor axonal polineuropathy with a widespread reduction of CMAPs especially in the bilateral median and peroneal nerves, associated to a much milder decrease of sensory nerve action potential (SNAP) amplitudes in the median, ulnar and sural nerves. EMG examination showed sporadic fasciculation potentials in the right anterior tibial and medial gastrocnemius muscles. MEPs obtained from ulnar and posterior tibial nerves as well as a 3-Tesla brain MRI were normal. A comprehensive neuropsychological battery did not show any cognitive impairment.

### Genetic testing

After informed consent, the mother, the son and two available unaffected relatives underwent blood sampling and genomic DNA extraction for genetic analyses. Whole exome sequencing was performed on DNA of the mother, her healthy brother and daughter and the affected son. By analyzing a virtual panel of genes associated with motor neuron disease, frontotemporal dementia and hereditary neuropathies, the affected probands were found to carry the heterozygous variant in the *MFN2* gene (reference sequence NM_014874.4) c.581A>C p (Asp194Ala). The variant is absent from population databases (e.g., gnomAD), reported as variant of unknown significance (VUS) in ClinVar (single submission) and classified as likely pathogenic according to the ACMG criteria PM1, PM2, PP2, PP3) (Richards et al., 2015).

Such variant segregated in the affected members of the family. *C9orf72* expansion was excluded in the probands. No other candidate variants were found in genes associated with ALS and FTD.

The ALS-FTD virtual gene panel that was analysed is reported in the Supplementary Information. Here, we also reported the list of genes associated with neurodegenerative diseases in which we searched for rare variants potentially modifying the phenotype.

## Discussion

We describe a mother and a son carrying a novel heterozygous mutation in the *MFN2* gene associated with a phenotypic heterogeneity. The p.(Asp194Ala) aminoacid substitution at the codon 194 falls in the large N-terminal GTPase domain, at the outer mitochondrial membrane in the cytosolic space. Such domain is the most functionally active site of the protein, being of critical importance for mitochondrial fusion, which in turn affects mitochondrial dynamics, distribution, quality control, and function; modulates endoplasmic reticulum - mitochondria tethering; axonal transport; calcium homeostasis. Altered MFN2 expression or function are associated with different pathological conditions. Several missense mutations have been reported in other CMT2 families, confirming the pathogenic role of variants in this mutational hotspot (Zhu et al., 2005; Cho et al., 2007). The mutations in the GTPase domain usually display high (but not always complete) penetrance in CMT2A.

The clinical course of the mother was suggestive of an ALS with FTD according to the Revised diagnostic criteria for ALS-FTD spectrum disorder (Strong et al., 2017). Moreover, she could also be diagnosed as a possible FTD, behavioural variant, according to Rascovsky criteria (Rascovsky et al., 2011). Conversely, the son had an earlier onset and a slow progression axonal neuropathy compatible with a diagnosis of CMT2A. Cases of CMT2A with both proximal and distal weakness, sometimes with a purely motor neuropathic phenotype were described (Abati et al., 2022). So far, only one patient with co-occurrence of CMT2A and ALS associated with a *MFN2* mutation has been reported, nevertheless the authors suggested a casual relationship (Marchesi et al., 2011). Our report may suggest that the association between *MFN2* and ALS may be causal rather than incidental. Furthermore, ours is the first description of an ALS-FTD phenotype related to a *MFN2* mutation.

The fast clinical deterioration with development of both proximal and distal muscle weakness, the presence of denervation activity and fasciculations in multiple districts, including the paravertebral one, associated to bulbar signs, psychiatric disorder and cognitive impairment, in accordance with the criteria of El Escorial (Ludolph et al., 2015), together with neuroimaging, strongly supported the diagnosis of ALS-FTD in the mother. On the other hand, the clinical, neuroimaging and neurophysiological data of the son would point towards a CMT2A polyneuropathy associated to mood disorder. We did not find alterations in other genes that can explain the different phenotypic expression between the two subjects. We can speculate that such different phenothype is partly due to the young age of the son, that deserves further clinical follow-up.

It is known that ALS and FTD are two diseases belonging to a broad spectrum of neurodegenerative disorders, often overlapping at genetic and clinical levels (Abramzon et al., 2020). In fact, both disorders can be present within the same family or even within the same individual. Several studies carried out in recent years have shown that up to 50% of patients with ALS develop cognitive impairment associated with FTD, as well as up to 30% of patients with FTD develop motor problems (Burrell et al., 2011; Turner et al., 2017).

In this regard, a growing body of evidence has demonstrated the progress of genetics in identifying the overlap of these two neurodegenerative conditions (Table 1) (Gentile et al., 2019).

**TABLE 1:** A review table describing the most genes associated with axonal CMT/ALS phenotypes. GARS, Glycyl-tRNA synthetase; FIG4, FIG4 phosphoinositide 5-phosphatase; KIF5A, Kinesin family member 5; NEFH, Neurofilament heavy; PLEKHG5, Pleckstrin homology and RhoGEF domain containing G5; VCP, Valosin containing protein; MFN2, Mitofusin 2; DYNC1H1, Dynein cytoplasmic 1 heavy chain 1; SPG11, Spatacsin vesicle trafficking associated. ALS, amyotrophic lateral sclerosis; CMT, charcot marie tooth; FTD, frontotemporal dementia; SMA, spinal muscular atrophy; SPG, spastic paraplegia; HMSN, hereditary motor and sensory neuropathy; dHMN, distal hereditary motor neuropathy; UPS, ubiquitin proteasome system IBMPFD, inclusion body myopathy paget disease frontotemporal dementia.

Genes	Chromosome	Inheritance	Disease
CHCHD10	22q11.23		CMT2A, ALS-FTD
GARS	7 p15	AD	CMT2D - dHMNVA - ALS
FIG4	6q21	AR-AD	ALS- CMT 4J
KIF5A	12q13	AD	CMT2 - SPG10 -ALS
NEFH	22q12	AD-AR	ALS - CMT2CC
PLEKHG5	1p36	AR	CMTC; SMA - SMA distal4 - ALS
VCP	9p13	AD	IBMPFD- ALS14 with or without FTD - CMT2Y
MFN2	1p36	AD-AR	CMT2A2A - CMT2A2B - dHMNVIA - ALS like-ALS-FTD
DYXC1H1	14q32	AD	CMT20 - dSMA1 -ALS
SPG11	15q21	AR	SPG11 - ALS5 juvenile - CMT2X

TARDBP, SQSTM1, VCP, FUS, TBK1, MAPT, GRN, CHCHD10 and especially C9orf72 are recognized as critical genetic players in the development of ALS-FTD spectrum disorders (Burrell et al., 2011; Gentile et al., 2019). In particular, missense changes in the mitochondrial protein CHCHD10 have been detected in few patients presenting heterogenous and complex clinical phenotypes including ALS-FTD (Ronchi et al., 2015; Gentile et al., 2019), pure ALS, flail-arm syndrome (Bannwarth et al., 2014) and CMT2 (Auranen et al., 2015). Altered mitochondrial cristae structure, impaired stress response and/or hampered mitochondrial dynamics (although in absence of changes in the levels of canonical fusion and fission proteins including MFN2) have been observed in patients, cellular and animal models harboring *CHCHD10* molecular defects (Shammas et al., 2023).

In this context, mitochondrial dysfunction has long been demonstrated as a common prominent early pathological feature of a variety of common neurodegenerative diseases, including Alzheimer’s disease (AD), Parkinson’s disease (PD) and ALS (GaoWangLiu et al., 2017).

The role of mitochondrial dynamics in the pathogenesis of neurodegenerative diseases has been increasingly confirmed by the interaction between different key regulators of fission and fusion, including mitofusins (GaoWangLiu et al., 2017).

Mutations of mitochondrial fusion and fission regulators including *MFN2* lead to mitochondrial depletion in neurites and synapses up to a total depletion in spinal dendrites with synaptic loss of motor neurons in mouse models (Mou et al., 2021). Consistently, the presence of inclusion bodies with TDP-43 representing the major component, has been demonstrated in damaged motor neurons of ALS patients (Neumann et al., 2006; Mou et al., 2021). A decrease of MFN2 has been recently described in fibroblasts upon progranulin silencing, supporting a link between *MFN2* and other genes related to the ALS-FTD spectrum. Russell et al. (Russell et al., 2013) showed that twenty-one distinct mutations in MFN2 identified in sporadic ALS patients render the protein defective in rescuing morphological defects in MFN2 knockout mouse embryonic fibroblasts (MEFs.) Moreover, the overexpression of TDP-43 preserved MFN2 levels upon transient progranulin silencing in fibroblasts of healthy controls (Gaweda-Walerych et al., 2021).

These data support the hypothesis that the novel *MFN2* variant may be responsible of such a novel phenotype, probably rare in the spectrum of MFN2-related disorders, but certainly peculiar and deserving further genetic studies.

## Conclusion

In conclusion, our findings support the clinical relevance of a novel *MFN2* mutation, firstly detected in two members from the same family, showing a different clinical presentation. Moreover, we reported the first case of ALS-FTD associated with a *MFN2* mutation, further expanding the spectrum of MFN-related disorders.

Future studies in large ALS-FTD cohorts will be needed to confirm the relevance of MFN2 as a further gene responsible for ALS-FTD disorder.

## Data Availability

The raw data supporting the conclusion of this article will be made available by the authors, without undue reservation.
