# Supplementary material for: Case report: Asp194Ala variant in MFN2 is associated with ALS-FTD in an Italian family
Source: Front Genet. 2023 Jul 20;14:1235887. doi: 10.3389/fgene.2023.1235887 (PMC10400291; doi:10.3389/fgene.2023.1235887)
Supplement: Supplementary file 1 [file Presentation1.pdf]

Supplementary Information

Virtual panel including gene associated with ALS-FTD

ALS2,ABCA7,ANG,APOE,APP,ATL1,BSCL2,CHCHD10,CHMP2B,CSF1R,DCTN1,FIG4,FUS,GBE1,GRN,HEXA,HNRNPA1,HSPD1,KIAA0196,KIF5A,MAPT,MATR3,OPTN,PFN1,PRF1,PRNP,PSEN1,PSEN2,REEP1,RNF216,SETX,SIGMAR1,SLC52A2,SLC52A3,SNCA,SOD1,SORL1,SPAST,SPG11,SPG20,SQSTM1,TARDBP,TIA1,TREM2,TUBA4A,UBE3A,UBQLN2,VAPB,VCP

Virtual panel of genes associated with neurodegenerative disorders

Gene Symbol	Phenotypes
ABCD1	Hereditary spastic paraplegia, MONDO:0019064;adrenal failure;VLCFA accumulation;spastic paraparesis
AFG3L2	Spinocerebellar ataxia 28, OMIM:610246;Ataxia, spastic, 5, autosomal recessive, OMIM:614487;Dystonia
ALS2	Amyotrophic lateral sclerosis 2, juvenile, OMIM:205100;Primary lateral sclerosis, juvenile, OMIM:606353
ANG	Amyotrophic lateral sclerosis 9, 611895
ANXA11	Amytrophic lateral sclerosis 23, OMIM:617839
APP	Alzheimer disease 1, familial, OMIM:104300;Cerebral amyloid angiopathy, Dutch, Italian, Iowa, Flemish, Arctic variants, OMIM:605714
ARSA	Metachromatic leukodystrophy, OMIM:250100;Dystonia
ATP13A2	Kufor-Rakeb syndrome, OMIM:606693;Dystonia;Spastic paraplegia 78, autosomal recessive, OMIM:617225
ATP1A3	ALTERNATING HEMIPLEGIA OF CHILDHOOD 2, OMIM:614820;CAPOS syndrome, OMIM:601338;DYSTONIA 12, OMIM:128235;Rapid-Onset Dystonia-Parkinsonism
ATP7B	Wilson disease, OMIM: 277900;Dystonia
AUH	Dystonia
C19orf12	Spastic paraplegia 43, autosomal recessive, OMIM:615043;Neurodegeneration with brain iron accumulation 4, OMIM: 614298
CACNA1G	Spinocerebellar ataxia 42, OMIM:616795
CCNF	Frontotemporal dementia and/or amyotrophic lateral sclerosis 5, OMIM:619141
CHCHD10	Myopathy, isolated mitochondrial, autosomal dominant, OMIM:616209
CHCHD2	Parkinson disease 22, autosomal dominant, OMIM:616710
CHMP2B	Frontotemporal dementia and/or amytrophic lateral sclerosis 7, OMIM:600795;Dystonia
CLCN2	Leukoencephalopathy with ataxia, OMIM:615651
CLN6	Ceroid lipofuscinosis, neuronal, 6, OMIM:601780;Ceroid lipofuscinosis, neuronal, Kufs type, adult onset, OMIM:204300
COASY	COASY protein-associated neurodegeneration;Neurodegeneration with brain iron accumulation 6, OMIM:615643
COL4A1	Brain small vessel disease with or without ocular anomalies, OMIM:175780;Angiopathy, hereditary, with nephropathy, aneurysms, and muscle cramps, OMIM:611773;Microangiopathy and leukoencephalopathy, pontine, autosomal dominant, OMIM:618564;{Hemorrhage, intracerebral, susceptibility to}, OMIM:614519
COL4A2	Brain small vessel disease 2, OMIM:614483;{Hemorrhage, intracerebral, susceptibility to}, OMIM:614519
CP	Dystonia;Cerebellar ataxia, OMIM:604290;Hemosiderosis, systemic, due to aceruloplasminemia, OMIM:604290
CSF1R	dementia, motor dysfunction (can include spasticity, ataxia, and parkinsonism) and epilepsy;Leukoencephalopathy, diffuse hereditary, with spheroids, OMIM:221820
CTSA	Cathepsin A-related arteriopathy-strokes-leukoencephalopathy, MONDO:0035551
CTSF	Ceroid lipofuscinosis, neuronal, 13, Kufs type, OMIM:615362
CYP27A1	Cerebrotendinous xanthomatosis, OMIM:213700;progressive lower extremity spasticity,often disproportionate to any degree of weakness

CYP7B1	Spastic paraplegia 5A, autosomal recessive, OMIM:270800
DARS2	Leukoencephalopathy with brain stem and spinal cord involvement and lactate elevation, OMIM:611105
DCTN1	Neuropathy, distal hereditary motor, type VIIB, OMIM:607641;Perry syndrome, OMIM:168605;{Amyotrophic lateral sclerosis, susceptibility to}, OMIM:105400
DNAJC5	Ceroid lipofuscinosis, neuronal, 4, Parry type, OMIM:162350
DNAJC6	Parkinson disease 19b, early-onset, OMIM:615528;Parkinson disease 19a, juvenile-onset, OMIM:615528
DNMT1	Cerebellar ataxia, deafness, and narcolepsy, autosomal dominant, OMIM:604121
EIF2B1	Leukoencephalopathy with vanishing white matter, OMIM:603896
EIF2B2	Leukoencephalopathy with vanishing white matter, OMIM:603896
EIF2B3	Leukoencephalopathy with vanishing white matter, OMIM:603896
EIF2B4	Leukoencephalopathy with vanishing white matter, OMIM:603896
EIF2B5	Leukoencephalopathy with vanishing white matter, OMIM:603896
ELOVL4	Spinocerebellar ataxia 34, OMIM:133190
EPM2A	Epilepsy, progressive myoclonic 2A (Lafora), OMIM:254780
ERBB4	Amyotrophic lateral sclerosis 19 OMIM:615515;amyotrophic lateral sclerosis type 19 MONDO:0014223
FBXO7	Dystonia;Parkinson disease 15, autosomal recessive, OMIM:260300
FTL	Neurodegeneration with brain iron accumulation 3, OMIM:606159
FUS	Amyotrophic lateral sclerosis 6, with or without frontotemporal dementia, OMIM:608030
GCH1	Dystonia, DOPA-responsive, with or without hyperphenylalaninemia, OMIM:128230;Hyperphenylalaninemia, BH4-deficient, B, OMIM:233910;Spastic paraplegia
GFAP	Autosomal Dominant Ataxia;Alexander disease, OMIM:203450
GLA	Fabry disease, OMIM:301500
GRN	Frontotemporal lobar degeneration with ubiquitin-positive inclusions, OMIM:607485;Aphasia, primary progressive, OMIM:607485;Ceroid lipofuscinosis, neuronal, 11, OMIM:614706
GSN	Amyloidosis, Finnish type, OMIM:105120;Finnish type amyloidosis, MONDO:0007097
HEXA	GM2-gangliosidosis, several forms, OMIM:272800;Tay-Sachs disease, OMIM:272800
HEXB	Sandhoff disease, infantile, juvenile, and adult forms, OMIM:268800
HNRNPA1	?Inclusion body myopathy wtih early-onset Paget disease without frontotemporal dementia type 3, OMIM:615424, Amyotrophic lateral sclerosis 20, OMIM:615426
HTRA1	dementia (disease), MONDO:0001627;CARASIL syndrome, OMIM:600142;Cerebral arteriopathy, autosomal dominant, with subcortical infarcts and leukoencephalopathy, type 2, OMIM:616779
ITM2B	Dementia, familial British, OMIM:176500;ABri amyloidosis, MONDO:0008306;Dementia, familial Danish, OMIM:117300;ADan amyloidosis, MONDO:0007297
KCNC3	Spinocerebellar ataxia 13, OMIM:605259
KCND3	Spinocerebellarataxia19, OMIM:607346
KIAA1161	Basal ganglia calcification, idiopathic, 7, autosomal recessive, OMIM:618317
KIF5A	Spastic paraplegia 10, autosomal dominant, OMIM:604187
LAMB1	Lissencephaly 5, OMIM:615191;cobblestone lissencephaly without muscular or ocular involvement, MONDO:0014077
LRRK2	LRRK2 G2019S mutation;{Parkinson disease 8}, OMIM:607060
LYST	Chediak-Higashi syndrome, OMIM:214500;peripheral neuropathy;Parkinsonism;spastic paraplegia
MAPT	Dementia, frontotemporal, with or without parkinsonism, OMIM:600274;Tauopathy and r;Supranuclear palsy, progressive, 601104;clinical presentation suggestive of cortico-basal/PSP syndrome;PARKINSON-DEMENTIA SYNDROME;{Parkinson disease, susceptibility to}, 168600;Pick disease, 172700;Clinical syndrome FTLD (Frontotemporal lobar degeneration);Supranuclear palsy, progressive atypical, 260540
NHLRC1	Epilepsy, progressive myoclonic 2B (Lafora), OMIM:254780
NOTCH3	Cerebral arteriopathy with subcortical infarcts and leukoencephalopathy 1, OMIM:125310

NPC1	Niemann-Pick disease, type C1, OMIM:257220;Niemann-Pick disease, type D, OMIM:257220
NPC2	Dystonia;Niemann-Pick disease, type C2, OMIM:607625
OPTN	Amyotrophic lateral sclerosis 12 with or without frontotemporal dementia, OMIM:613435
PANK2	Dystonia;Neurodegeneration with brain iron accumulation 1, OMIM:234200
PARK7	Parkinson disease 7, autosomal recessive early-onset, OMIM:606324
PDGFB	Basal ganglia calcification, idiopathic, 5, OMIM:615483
PDGFRB	Dystonia;Basal ganglia calcification, idiopathic, 4, OMIM:615007
PFN1	Amyotrophic lateral sclerosis 18, OMIM:614808
PINK1	Parkinson disease 6, early onset, OMIM:605909;Dystonia
PLA2G6	Parkinson disease 14, autosomal recessive, OMIM:612953;Neurodegeneration with brain iron accumulation 2B, OMIM:610217
PRKN	Parkinson disease, juvenile, type 2, OMIM:600116;Dystonia
PRNP	Creutzfeldt-Jakob disease, OMIM:123400;Huntington disease-like 1, OMIM:603218;Dementia;Gerstmann-Straussler disease, OMIM:137440
PSEN1	Alzheimer disease, type 3, with spastic paraparesis and unusual plaques, OMIM:607822;Alzheimer disease, type 3, with spastic paraparesis and apraxia, OMIM:607822;Dystonia;Dementia, frontotemporal, OMIM:600274;Pick disease, OMIM:172700;Alzheimer disease, type 3, OMIM:607822
PSEN2	Alzheimer disease-4, OMIM:606889;Alzheimer disease 4, MONDO:0011743
RNF216	Cerebellar ataxia and hypogonadotropic hypogonadism, OMIM:212840
SETX	Amyotrophic lateral sclerosis 4, juvenile, OMIM:602433
SLC20A2	Dystonia;Basal ganglia calcification, idiopathic, 1, OMIM:158378
SNCA	Parkinson disease 4, OMIM:605543;Parkinson disease 1, OMIM:168601;Dementia, Lewy body, OMIM:127750
SOD1	Amyotrophic lateral sclerosis 1, OMIM:105400
SPAST	Spastic paraplegia 4, autosomal dominant, OMIM:182601;hereditary spastic paraplegia 4, MONDO:0008438
SPG11	early onset parkinsonism, levo dopa responsve;Spastic paraplegia 11, autosomal recessive, OMIM:604360;Complex parkinsonism;hereditary spastic paraparesis;Amyotrophic lateral sclerosis 5, juvenile, OMIM:602099
SQSTM1	Frontotemporal dementia and/or amyotrophic lateral sclerosis 3, OMIM:616437
STUB1	Autosomal recessive spinocerebellar ataxia type 16, OMIM:615768;autosomal recessive spinocerebellar ataxia 16, MONDO:0014339;Spinocerebellar ataxia 48, OMIM:618093;spinocerebellar ataxia 48, MONDO:0032526
SYNJ1	Parkinson disease 20, early-onset, OMIM:615530
TARDBP	Amyotrophic lateral sclerosis 10, with or without FTD, OMIM:612069
TBK1	Frontotemporal dementia and/or amyotrophic lateral sclerosis 4, OMIM:616439
TMEM240	Spinocerebellar ataxia 21, OMIM:607454
TREM2	Polycystic lipomembranous osteodysplasia with sclerosing leukoencephalopathy 2, OMIM:618193;Dystonia
TREX1	Aicardi-Goutieres syndrome 1, dominant and recessive, OMIM:225750;Vasculopathy, retinal, with cerebral leukoencephalopathy and systemic manifestations, OMIM:192315
TTC19	Mitochondrial complex III deficiency, nuclear type 2, OMIM:615157
TTR	Amyloidosis, hereditary, transthyretin-related, OMIM:105210;Carpal tunnel syndrome, familial, OMIM:115430
TYROBP	Polycystic lipomembranous osteodysplasia with sclerosing leukoencephalopathy 1, OMIM:221770
UBQLN2	Amyotrophic lateral sclerosis 15, with or without frontotemporal dementia, OMIM:300857
VAPB	Amyotrophic lateral sclerosis 8, OMIM:608627
VCP	Frontotemporal dementia and/or amyotrophic lateral sclerosis 6, OMIM:613954
VPS13A	Choreoacanthocytosis, OMIM:200150
VPS35	{Parkinson disease 17}, OMIM:614203
WDR45	Dystonia;Neurodegeneration with brain iron accumulation 5, OMIM:300894

XK	McLeod syndrome with or without chronic granulomatous disease, OMIM:300842;McLeod neuroacanthocytosis syndrome, MONDO:0018945
XPR1	Basal ganglia calcification, idiopathic, 6, OMIM:605237
AP5Z1	Spastic paraplegia 48, autosomal recessive, OMIM:613647
ARHGEF28	Amyotrophic lateral sclerosis, MONDO:0004976
ATP2B3	?Spinocerebellar ataxia, X-linked 1, OMIM:302500
ATP6AP2	?Parkinsonism with spasticity, X-linked, OMIM:300911;Mental retardation, X-linked, syndromic, Hedera type, OMIM:300423
CCDC88C	?Spinocerebellar ataxia 40, OMIM:616053
CIZ1	Dystonia 23, MONDO:0013928
COQ2	{Multiple system atrophy, susceptibility to}, OMIM:146500
CST3	Cerebral amyloid angiopathy, OMIM:105150
DAO	Amyotrophic lateral sclerosis, MONDO:0004976
DNAJC13	
EIF4G1	{Parkinsons disease 18}, OMIM:614251
EWSR1	Amyotrophic lateral sclerosis, MONDO:0004976
GBA	{Parkinson disease, late-onset, susceptibility to}, OMIM:168600;Gaucher disease, type I, OMIM:230800
GCDH	Dystonia;Glutaricaciduria, type I, OMIM:231670
GIGYF2	{Parkinson disease 11}, OMIM:607688
HNRNPA2B1	Amyotrophic lateral sclerosis, MONDO:0004976;?Inclusion body myopathy with early-onset Paget disease with or without frontotemporal dementia 2, OMIM:615422
MARS2	Spastic ataxia 3, autosomal recessive, OMIM:611390
MATR3	Amyotrophic lateral sclerosis 21, OMIM:606070
NEK1	{Amyotrophic lateral sclerosis, susceptibility to, 24}, OMIM:617892;amyotrophic lateral sclerosis, susceptibility to, 24, MONDO:0054750
NR4A2	Intellectual developmental disorder with language impairment and early-onset DOPA-responsive dystonia-parkinsonism, OMIM:619911
PRKRA	Dystonia 16, OMIM:612067
PRPH	{Amyotrophic lateral sclerosis, susceptibility to}, OMIM:170710
SIGMAR1	?Amyotrophic lateral sclerosis 16, juvenile, OMIM:614373
SLC30A10	Hypermanganesemia with dystonia 1, OMIM:613280
SNCB	Dementia, Lewy body, OMIM:127750
SORL1	Alzheimer's Disease
SS18L1	Amyotrophic lateral sclerosis, MONDO:0004976
TAF1	Dystonia-Parkinsonism, X-linked, OMIM:314250
TUBA4A	Amyotrophic lateral sclerosis 22 with or without frontotemporal dementia, OMIM:616208
TUBB4A	Leukodystrophy, hypomyelinating, 6, OMIM:612438;Dystonia 4, torsion, autosomal dominant, OMIM:128101
UQCRC1	Parkinsonism with polyneuropathy, OMIM:619279
VPS13C	Parkinson disease 23, autosomal recessive, early onset, OMIM:616840
AAAS	Achalasia-addisonianism-alacrimia syndrome, OMIM:231550;Triple-A syndrome, MONDO:0009279
AARS	Charcot-Marie-Tooth disease, axonal, type 2N, OMIM:613287;Charcot-Marie-Tooth disease axonal type 2N, MONDO:0013212
ABCB7	Anemia, sideroblastic, with ataxia,;Sideroblastic Anemia and Ataxia
ABHD12	Polyneuropathy, Hearing Loss, Ataxia, Retinitis Pigmentosa and Cataract (PHARC);Polyneuropathy, hearing loss, ataxia, retinitis pigmentosa, and cataract
ACTB	Dystonia, juvenile-onset, 607371Baraitser-Winter syndrome 1, 243310

ADAR	Aicardi-Goutieres syndrome 6, OMIM:615010
ADCY5	dystonia;Familial dyskinesia 606703;Dyskinesia, familial, with facial myokymia, 606703
AIMP1	Leukodystrophy, hypomyelinating, 3, 260600
ALAS2	
ALDH18A1	Spastic paraplegia 9A, autosomal dominant;ADCL3 AUTOSOMAL RECESSIVE MENTAL RETARDATION-JOINT HYPERMOBILITY-SKIN LAXITY WITH OR WITHOUT METABOLIC ABNORMALITIES (MRJHSL) SPASTIC PARAPLEGIA 9, AUTOSOMAL DOMINANT;Spastic paraplegia 9B, autosomal recessive CUTIS LAXA, AUTOSOMAL DOMINANT 3;SPG9
AMPD2	Spastic paraplegia homozygous frameshift reported in single family (Novarino et al, 2014).;Hereditary Spastic Paraplegia?;Pontocerebellar hypoplasia 9 (#615809);Pontocerebellar hypolplasia (biallelic)
ANO10	Spinocerebellar ataxia, autosomal recessive 10, 613728
ANO3	Dystonia 24, 615034;familial form of cranio-cervical dystonia
AP1S2	Pettigrew syndrome, OMIM:304340
AP4B1	Spastic paraplegia 47, autosomal recessive, OMIM:614066;Hereditary spastic paraplegia 47, MONDO:0013551
AP4E1	Spastic paraplegia 51, autosomal recessive, OMIM:613744;Hereditary spastic paraplegia 51, MONDO:0013401
AP4M1	Spastic paraplegia 50, autosomal recessive
AP4S1	developmental delay;seizures;Spastic paraplegia 52, autosomal recessive
APTX	Ataxia with Oculomotor Apraxia;Ataxia, early-onset, with oculomotor apraxia and hypoalbuminemia;Dystonia
AR	Spinal and bulbar muscular atrophy of Kennedy, OMIM:313200
ARG1	Argininemia, OMIM:207800
ARL6IP1	
ARSI	
ARX	Dystonia
ATCAY	Ataxia, cerebellar, Cayman type;Cerebellar Ataxia, Cayman type
ATL1	Spastic paraplegia 3A, autosomal dominant,;Spastic Paraplegia, Dominant;Spastic paraplegia 3A, autosomal dominant
ATM	Ataxia-telangiectasia, OMIM:208900
ATN1	Dentatorubral-pallidoluysian atrophy, OMIM:125370
ATP1A2	Dystonia;alternating hemiplegia of childhood 104290;familial basilar migraine 602481;migraine;familial hemiplegic migraine type 2, 602481
ATP8A2	
ATXN1	Spinocerebellar ataxia 1, OMIM:164400
ATXN10	Spinocerebellar ataxia 10, OMIM:603516
ATXN2	Spinocerebellar ataxia 2, OMIM:183090;{Amyotrophic lateral sclerosis, susceptibility to, 13}, OMIM:183090;{Parkinson disease, late-onset, susceptibility to}, OMIM:168600
ATXN3	Machado-Joseph disease, OMIM:109150;Susceptibility to Late-Onset Parkinson Disease
ATXN7	Spinocerebellar ataxia 7, OMIM:164500
ATXN8	Spinocerebellar ataxia 8 608768
B4GALNT1	Spastic paraplegia 26, autosomal recessive
BCAP31	Deafness, dystonia and cerebellar hypomyelination, 300475
BEAN1	Spinocerebellar ataxia 31 117210
BSCL2	Silver spastic paraplegia syndrome, OMIM:270685
C12orf65	Spastic paraplegia 55, autosomal recessive, OMIM:615035
C9orf72	Frontotemporal dementia and/or amyotrophic lateral sclerosis 1, OMIM:105550
CA8	Cerebellar ataxia and mental retardation with or without quadrupedal locomotion 3
CACNA1A	Spinocerebellar ataxia 6, OMIM:183086

CACNB4	Episodic ataxia, type 5;EPILEPSY, IDIOPATHIC GENERALIZED, SUSCEPTIBILITY TO, 9;EPISODIC ATAXIA, TYPE 5;Episodic Ataxia
CAMTA1	Cerebellarataxia, nonprogressive, with mental retardation, 614756
CAPN1	Spastic paraplegia 76 autosomal recessive 616907
CASK	FG syndrome 4, 300422;Mental retardation and microcephaly with pontine and cerebellar hypoplasia, 300749
CCT5	Neuropathy, hereditary sensory, with spastic paraplegia;Sensory Neuropathy with Spastic Paraplegia
CDK16	Intellectual disability and spastic paraplegia
CHMP1A	Pontocerebellar hypoplasia, type 8, 614961
1 CLP	Pontocerebellar hypoplasia 10, 615803
COG5	Congenital disorder of glycosylation, type IIi 613612
COQ8A	Coenzyme Q10 deficiency, primary 4, 612016;Spinocerebellar Ataxia Type
COX20	Mitochondrial complex IV deficiency, 220110
CSTB	Epilepsy, progressive myoclonic 1A (Unverricht and Lundborg), OMIM:254800
CWF19L1	Spinocerebellar ataxia, autosomal recessive 17, 616127
CYP2U1	Autosomal recessive spastic paraplegia 56 (#615030) complex form of disorder, ataxia not yet identified in affected patients.;Spastic paraplegia 56, autosomal recessive
DAB1	Spinocerebellar ataxia 37 615945
DARS	Brain stem and spinal cord Hypomyelination;leg spasticity;Hypomyelination with brainstem and spinal cord involvement and leg spasticity, 615281
DCAF17	Dystonia;Woodhouse-Sakati syndrome
DDC	Aromatic L-amino acid decarboxylase deficiency, OMIM:608643;Aromatic L-amino acid decarboxylase deficiency, MONDO:0012084
DDHD1	Spastic paraplegia 28, autosomal recessive
DDHD2	Autosomal recessive paraplegia 54 (#615033). Complex form of disease ataxia reported amongst the phenotypic features in Citterio et al. (2014), Journal of Neurology, 261, pp.373-381 and Doi et al. (2014), Scientific Reports, 4, 7132.;Spastic paraplegia 54, autosomal recessive
DLAT	Dystonia
DMXL2	Sensorineural Hearing Loss;ORPHA90636;OMIM:612186
DNAJC19	3-methylglutaconic aciduria, type V 610198;dilated cardiomyopathy with ataxia (DCMA) syndrome
DRD2	Dystonia, myoclonic, 159900
DRD5	{Blepharospasm, primary benign}, 606798
DSTYK	Spastic paraplegia 23, 270750
DYNC1H1	Charcot Marie Tooth, SMA, Intellectual disability
EARS2	Dystonia
ELOVL5	Spinocerebellar ataxia 36 (#615957)
ENTPD1	Spastic paraplegia 64, autosomal recessive, OMIM:615683
ERCC6	Dystonia
ERLIN1	Hereditary spastic paraplegia;Spastic paraplegia 62, 615681
ERLIN2	neurodegeneration;hereditary spastic paraplegia;Spastic paraplegia 18, autosomal recessive, 611225;Spastic paraplegia, autosomal dominant
EXOSC3	Pontocerebellar hypoplasia, type 1B, OMIM:614678
FA2H	Dystonia;fatty acid hydroxylase-associated neurodegeneration;Spastic paraplegia 35, autosomal recessive
FARS2	Spastic paraplegia 77, autosomal recessive, 617046
FASTKD2	Combined oxidative phosphorylation deficiency 44, OMIM:618855
FGF14	Spinocerebellar ataxia 27

FIG4	Amyotrophic lateral sclerosis 11 OMIM:612577;amyotrophic lateral sclerosis type 11 MONDO:0012945;Charcot-Marie-Tooth disease, type 4J, OMIM:611228;Charcot-Marie-Tooth disease type 4J MONDO:0012640
FLVCR1	Posterior Column Ataxia with Retinitis Pigmentosa;Ataxia, posterior column, with retinitis pigmentosa,
FMR1	Fragile X syndrome, OMIM:300624;Fragile X tremor/ataxia syndrome, OMIM:300623
FOLR1	Neurodegeneration due to cerebral folate transport deficiency, 613068
FOXG1	Dystonia
FOXRED1	Dystonia
FXN	Friedreich ataxia, OMIM:229300;Friedreich ataxia with retained reflexes, OMIM:229300
GAD1	Cerebralpalsy,spasticquadriplegic,1,603513
GAMT	Dystonia
GBA2	Spastic paraplegia 46, autosomal recessive, 614409
GJC2	Leukodystrophy, hypomyelinating, 2;Autosomal Recessive Ataxia;Spastic paraplegia 44, autosomal recessive
GLRA1	Hyperekplexia, hereditary 1, 149400
GLRB	Hyperekplexia 2, 614619
GNAL	adult-onset cranio-cervical dystonia;Dystonia 25, 615073
GNAO1	Neurodevelopmental disorder with involuntary movements, 617493
GOSR2	Epilepsy, progressive myoclonic 6, 614018
GPAA1	Glycosylphosphatidylinositol biosynthesis defect 15, 617810
GRID2	Spinocerebellar ataxia, autosomal recessive 18, 616204
GRM1	Spinocerebellar ataxia 44, OMIM:617691
HACE1	Spastic paraplegia;psychomotor retardation;seizure;Spastic paraplegia and psychomotor retardation with or without seizures, 616756
HFE	
HPCA	Dystonia 2, torsion, autosomal recessive, 224500;generalized dystonia with additional neurological features;adolescence-onset segmental dystonia;childhood-onset generalized dystonia
HPRT1	Lesch-Nyhan syndrome, OMIM:300322
HSPD1	Spastic paraplegia 13, autosomal dominant, OMIM:605280
HTRA2	Parkinson Disease, Dominant;Parkinson disease 13, 610297;3-methylglutaconic aciduria, type VIII 617248
HTT	Huntington disease, OMIM:143100
IBA57	?Spastic paraplegia 74, autosomal recessive, OMIM:616451
IPPK	Early Onset Complex Disease
ITPR1	Spinocerebellar ataxia 29;Spinocerebellar ataxia 15
JPH3	Huntington disease-like 2, OMIM:606438
KCNA1	myokymia with periodic ataxia;Episodic ataxia/myokymia syndrome,;EPISODIC ATAXIA, TYPE 1
KCNJ10	Seizures, Sensorineural Deafness, Ataxia, Mental Retardation, and Electrolyte Imbalance Syndrome
KCNK18	MIGRAINE, WITH OR WITHOUT AURA, SUSCEPTIBILITY TO, 13
KCNQ2	Dystonia;Myokymia, 121200
KCNQ3	Seizures, benign neonatal, type 2, 121201
KDM5C	Intellectual developmental disorder, X-linked syndromic, Claes-Jensen type, OMIM:300534
KIDINS220	Spastic paraplegia, intellectual disability, nystagmus, and obesity, OMIM:617296
KIF1A	Spastic paraplegia 30, autosomal dominant, OMIM:610357;Spastic paraplegia 30, autosomal recessive, OMIM:610357;NESCAV syndrome, OMIM:614255
KIF1C	Spastic ataxia 2, autosomal recessive, OMIM:611302
KLC4	spastic paraplegia;progressive complicated spastic paraplegia
KMT2B	early-onset dystonia

L1CAM	X-linked hydrocephalus, MASA syndrome, Hereditary spastic paraplegia
L2HGDH	Dystonia
MAG	Spastic paraplegia 75, autosomal recessive, OMIM:616680
MARS	
MAT1A	Dystonia
MCOLN1	Dystonia
MECR	Dystonia, childhood-onset, with optic atrophy and basal ganglia abnormalities 617282
MMACHC	Ataxia and hypogonadism;Methylmalonic aciduria and homocystinuria, cblC type, 277400
MMADHC	Dystonia
MPV17	Dystonia
MR1	Dystonia;Paroxysmal/Episodic dystonia
MRE11	Ataxia-telangiectasia-like disorder;Ataxia-Telangiectasia-Like Disorder
MT-ATP6	Neuropathy, Ataxia, and Retinitis Pigmentosa
MT-ND6	Leber Optic Atrophy And Dystonia
MTPAP	Ataxia, spastic, 4,;Ataxia, spastic, 4;Spastic ataxia 4, autosomal recessive
MTTP	Abetalipoproteinemia, 200100
MVK	Mevalonic aciduria, OMIM:610377
NAGLU	Sensory neuropathy turning into a mild sensory ataxia (AD). Also Sanfilippo syndrome B (AR) (OMIM #252920)
NDUFA12	Mitochondrial complex I deficiency, nuclear type 23, OMIM:618244
NEFH	susceptibility to amyotrophic lateral sclerosis (ALS)
NIPA1	Spastic paraplegia 6, autosomal dominant;Spasticparaplegia6,autosomaldominant,600363
NKX2-1	Chorea, hereditary benign 118700;Choreoathetosis, hypothyroidism, and neonatal respiratory distress 610978
NKX6-2	Spastic ataxia 8, autosomal recessive, with hypomyelinating leukodystrophy 617560
NOP56	Spinocerebellar ataxia 36, OMIM:614153
NT5C2	Spastic paraplegia 45, autosomal recessive, 613162
OPA3	Costeff syndrome;3-methylglutaconic aciduria, type III, 258501
OPHN1	Mental retardation, X-linked, with cerebellar hypoplasia and distinctive facial appearance, 300486
PAX2	Ataxia,spastic2,autosomalrecessive(2)
PAX6	Aniridia, Cerebellar Ataxia, And Mental Retardation
PCDH12	microcephaly;intellectual disability;perithalamic hyperechogenicity;hypothalamic abnormalities;periventricular hyperechogenicity;epilepsy;midbrain abnormalities
PCLO	Pontocerebellar hypoplasia 3 homozygous non-sense variant identified in the affected individuals of a single pedigree.
PDHX	Dystonia
PDYN	Spinocerebellar ataxia 23
PEX16	Zellweger syndrome (614876);Peroxisome biogenesis disorder 8B (#614877) infantile progressive ataxia and spastic paresis
PGAP1	
PIK3R5	Ataxia-oculomotor apraxia 3
PLP1	Dystonia;Spastic paraplegia 2, X-linked
PMPCA	Non-progressive cerebellar ataxia recessive variants identified in 17 patients from four different families.
PNKD	Familial Paroxysmal Nonkinesigenic Dyskinesia;PAROXYSMAL NONKINESIGENIC DYSKINESIA 1;Paroxysmal nonkinesigenic dyskinesia, 118800
PNKP	Ataxia with oculomotor apraxia 4 (#616267)

PNPLA6	Spinocerebellar ataxia, hypogonadotropic hypogonadism and chorioretinal dystrophy (Boucher-Neuhauser syndrome, #215470);Spastic paraplegia 39, autosomal recessive;Oliver-McFarlane syndrome (#603197);Autosomal recessive spastic paraplegia 39 (#612020), ataxia seen in some patients
PNPT1	Dystonia
POLG	Mitochondrial recessive ataxia syndrome (includes SANDO and SCAE)
POLR3A	Leukodystrophy, hypomyelinating, 7, with or without oligodontia and/or hypogonadotropic hypogonadism;Autosomal Recessive Ataxia
PPP2R2B	Spinocerebellar ataxia 12, OMIM:604326
PRICKLE1	Progressive Myoclonus Epilepsy with Ataxia
PRKCG	Spinocerebellar ataxia 14
PRRT2	dystonia and occasionally hemiplegic migraine and epilepsy;episodic kinesigenic dyskinesia;EPISODIC KINESIGENIC DYSKINESIA 1;CONVULSIONS, FAMILIAL INFANTILE, WITH PAROXYSMAL CHOREOATHETOSIS;Paroxysmal kinesigenic choreoathetosis (PKD1) and infantile convulsions;SEIZURES, BENIGN FAMILIAL INFANTILE, 2;Episodic kinesigenic dyskinesia 1, 128200
PTEN	Dystonia
PTS	Dystonia
QDPR	Dystonia
RAB39B	early-onset parkinsonism and intellectual disability;Waisman syndrome, OMIM:311510
RAB3GAP2	Martsolf syndrome 1, OMIM:212720
RARS2	epilepsy;Pontocerebellar hypoplasia
REEP1	Spastic paraplegia 31, autosomal dominant
REEP2	Spastic paraplegia 72, autosomal recessive, 615625;?Spastic paraplegia 72, autosomal dominant,615625
RELN	
RNASEH2A	Dystonia
RNASEH2B	Aicardi-Goutieres syndrome 2, OMIM:610181;Dystonia (onset in infancy)
RNASEH2C	Dystonia
RNF170	Ataxia, sensory, 1, autosomal dominant
RTN2	Spastic paraplegia 12, autosomal dominant
RUBCN	
SACS	Spastic ataxia, Charlevoix-Saguenay type
SAMHD1	Dystonia
SAR1B	Chylomicron retention disease 246700
SCN1A	several epilepsy, convulsion and migraine disorders.;familial hemiplegic migraine 3;Dravet syndrome
SCN8A	epilepsy;Cognitive impairment with or without cerebellar ataxia, 614306;paroxysmal kinesigenic dyskinesias
SCN9A	Erythermalgia, primary, 133020;Epilepsy, generalized, with febrile seizures plus, type 7, 613863;Congenital Indifference to Pain;Paroxysmal Extreme Pain Disorder;Dysosteosclerosis;Insensitivity to pain, channelopathy-associated, 243000;Hereditary Sensory Neuropathy;Paroxysmal extreme pain disorder, 167400;Febrile seizures, familial, 3B, 613863;Erythermalgia, Primary
SCP2	Leukoencephalopathy with dystonia and motor neuropathy, 613724
SDHAF1	Dystonia
SEPSECS	Pontocerebellar hypoplasia type 2D (613811)
SERAC1	3-methylglutaconic aciduria with deafness, encephalopathy, and Leigh-like syndrome, OMIM:614739
SGCE	Myoclonus dystonia syndrome;Myoclonus-Dystonia;maternally imprinted Dystonia-11, myoclonic, 159900
SIL1	Marinesco-Sjogren syndrome, 248800
SLC16A2	Allan-Herndon-Dudley syndrome, OMIM:300523
SLC19A3	Dystonia
SLC1A3	EPISODIC ATAXIA, TYPE 6;Episodic ataxia, type 6,

SLC1A4	Spastic tetraplegia, thin corpus callosum, and progressive microcephaly, 616657
SLC25A46	Neuropathy, hereditary motor and sensory, type VIB 616505
SLC2A1	GLUT1 DEFICIENCY SYNDROME 1;dystonia 9;GLUT1 deficiency syndrome 1, infantile onset, severe;EPILEPSY, IDIOPATHIC GENERALIZED;Dystonia;GLUT1 deficiency syndrome 2, childhood onset;GLUT1 deficiency syndrome 1, 606777;paroxysmal exertion-induced dyskinesia with or without epilepsy and/or hemolytic anemia;GLUT1 deficiency syndrome 1;GLUT1 deficiency syndrome 2;spastic paraplegia
SLC33A1	Spastic paraplegia 42, autosomal dominant,
SLC39A14	Hypermanganesemia with dystonia 2 617013
SLC41A1	Parkinson disease (Yan (2011) Int J Neurosci 121,632)
SLC46A1	Dystonia
SLC52A1	Riboflavin deficiency (condition resembling childhood-onset motor neurone disease)
SLC52A2	Brown-Vialetto-Van Laere syndrome 2
SLC52A3	Fazio-Londe disease;Brown-Vialetto-Van Laere syndrome 1
SLC6A3	Parkinsonism-dystonia, infantile, 613135;{Nicotine dependence, protection against}, 188890
SLC6A5	Hyperekplexia 3, 614618
SLC9A6	Mental retardation, X-linked syndromic, Christianson type, 300243
SNCAIP	Parkinson Disease, Dominant/Recessive
SNX14	Autosomal recessive spinocerebellar ataxia (#616354)
SPART	Troyer syndrome, OMIM:275900
SPG21	Spastic Paraplegia, Recessive
SPG7	Spastic paraplegia 7 (#607259) complex forms of the disease. Actually associated with a range of phenotypes including adult-onset ataxia;Spastic paraplegia 7, autosomal recessive
SPR	paediatric form of dopa responsive dystonia;Dystonia, dopa-responsive, due to sepiapterin reductase deficiency, 612716;Dystonia, dopa-responsive, due to sepiapterin reductase deficiency 612716;Dopa-Responsive Dystonia
SPTBN2	Spinocerebellar ataxia 5, OMIM:600224;Spinocerebellar ataxia, autosomal recessive 14, OMIM:615386
SRD5A3	Congenital disorder of glycosylation, type Iq, 612379;Kahrizi syndrome, 612713
SUCLA2	Dystonia
SUOX	Dystonia
SYNE1	Cerebellar Ataxia;Spinocerebellar ataxia, autosomal recessive 8
SYT14	Spinocerebellarataxia,autosomalrecessive11,614229
TAF15	Amyotrophic lateral sclerosis
TBP	Spinocerebellar ataxia 17, OMIM:607136;{Parkinson disease, susceptibility to}, OMIM:168600
TDP1	Spinocerebellar ataxia, autosomal recessive with axonal neuropathy
TECPR2	Spastic paraplegia 49, autosomal recessive, 615031
TET2	
TFG	Spastic paraplegia 57, autosomal recessive, OMIM:615658
TGM6	Spinocerebellar ataxia 35, 613908
TH	Segawa syndrome;DOPA-responsive dystonia;infantile parkinsonism;Segawa syndrome, recessive, 605407;paediatric form of dopa responsive dystonia
THAP1	Dystonia;Dystonia 6, torsion, 602629;DYT6
TIA1	
TIMM8A	Deafness-Dystonia-Optic Neuronopathy Syndrome
TOR1A	Dystonia-1, torsion, OMIM:128100;Dystonic disorder, MONDO:0003441
TPK1	Dystonia
TPP1	Ceroid lipofuscinosis, neuronal, 2, 204500;Spinocerebellar ataxia, autosomal recessive 7, 609270

TSEN2	Pontocerebellar hypoplasia 2B, 612389
TSEN34	Pontocerebellar hypoplasia 2C (612390)
TSEN54	Pontocerebellar hypoplasia 2A, 277470;Pontocerebellar hypoplasia 4, 225753
TTBK2	Spinocerebellar ataxia 11
TTPA	Ataxia with isolated vitamin E deficiency;Ataxia with Vitamin E Deficiency
TWNK	Spinocerebellar Ataxia, Recessive;Ataxia Neuropathy Spectrum Disorders, Dominant;Progressive external ophthalmoplegia with mitochondrial DNA deletions, autosomal dominant 3, 609286;Perrault syndrome 5, 616138;Mitochondrial DNA depletion syndrome 7 (hepatocerebral type), 271245
UBR4	Episodic ataxia
UCHL1	?{Parkinson disease 5, susceptibility to};Early onset ataxia and optic neuropathy
UNC13A	
USP8	
VAC14	Striatonigral degeneration, childhood-onset 617054
VAMP1	Spastic ataxia 1, autosomal dominant, 108600
VEGFA	
VLDLR	Cerebellar hypoplasia and mental retardation with or without quadrupedal locomotion 1, 224050
VPS13D	Spinocerebellar ataxia, autosomal recessive 4, 607317
VPS37A	Dystonia;Spastic paraplegia 53, autosomal recessive
VPS53	Pontocerebellar hypoplasia 2E (#615851)
VRK1	Pontocerebellar hypoplasia 1A (#607596)
WASHC5	Spastic paraplegia 8, autosomal dominant
WDR45B	Neurodevelopmental disorder with spastic quadriplegia and brain abnormalities with or without seizures, OMIM:617977
WDR48	
WDR73	Galloway Mowat syndrome, when patients are ambulant ataxia is a recognised feature;Galloway-Mowat syndrome 1, 251300
WDR81	Cerebellar ataxia, mental retardation, and dysequilibrium syndrome 2, 610185
WFS1	Wolfram syndrome 1, 222300
WWOX	Autosomal recessive spinocerebellar ataxia 12, 614322
XRCC1	Spinocerebellar ataxia, autosomal recessive 26, OMIM:617633
YY1	Gabriele-de Vries syndrome 617557
ZEB2	
ZFYVE26	Spastic paraplegia 15, autosomal recessive;Autosomal recessive spastic paraplegia 15 (#270700) complex form of the disease including ataxia. Pyle et al. (2015), Brain, 138, pp.276-283. Implicated in undiagnosed ataxia.
ZFYVE27	Spastic paraplegia 33, autosomal dominant
ZNF592	Spinocerebellar ataxia, autosomal recessive 5
DNAJB2	Spinal muscular atrophy, distal, autosomal recessive, 5, 614881
DNAJC7	amyotrophic lateral sclerosis
GBE1	Polyglucosan body disease, adult form, OMIM:263570
GLT8D1	Amyotrophic lateral sclerosis
PSAP	Parkinson disease, AD
SPTLC1	Juvenile ALS
